# Noise Attenuation Performance of a Helmholtz Resonator Array Consist of Several Periodic Parts

**DOI:** 10.3390/s17051029

**Published:** 2017-05-04

**Authors:** Dizi Wu, Nan Zhang, Cheuk Ming Mak, Chenzhi Cai

**Affiliations:** 1 School of Civil Engineering, Central South University, Changsha 410083, China; diziwu@hotmail.com; 2 School of Architecture and Art, Central South University, Changsha 410083, China; zhangnan_csu@126.com; 3 Department of Building Services Engineering, The Hong Kong Polytechnic University, Hong Kong, China; cheuk-ming.mak@polyu.edu.hk

**Keywords:** Helmholtz resonator, noise attenuation, periodic structure, finite element method

## Abstract

The acoustic performance of the ducted Helmholtz resonator (HR) system is analyzed theoretically and numerically. The periodic HR array could provide a wider noise attenuation band due to the coupling of the Bragg reflection and the HR’s resonance. However, the transmission loss achieved by a periodic HR array is mainly dependent on the number of HRs, which restricted by the available space in the longitudinal direction of the duct. The full distance along the longitudinal direction of the duct for HR’s installation is sometimes unavailable in practical applications. Only several pieces of the duct may be available for the installation. It is therefore that this paper concentrates on the acoustic performance of a HR array consisting of several periodic parts. The transfer matrix method and the Bragg theory are used to investigate wave propagation in the duct. The theoretical prediction results show good agreement with the Finite Element Method (FEM) simulation results. The present study provides a practical way in noise control application of ventilation ductwork system by utilizing the advantage of periodicity with the limitation of available completed installation length for HRs.

## 1. Introduction

In modern buildings, the ventilation ductwork system plays a significant role in maintaining good indoor environment such as air quality, air temperature and air humidity [1,2]. However, the accompanied duct-borne noise generated by in-ducted dampers, sensors, duct corners, and other in-ducted elements can be a disturbance to humans [3,4,5]. To reduce the duct-borne noise in the ventilation ductwork system is therefore an important matter of attention. The Helmholtz resonator (HR) is widely and commonly used as an effective silencer in air duct noise control applications to reduce low-frequency noise at its resonance frequency with narrow attenuation band. It is easy to design a HR with a desired resonance frequency due to the fact its resonance frequency is only determined by the physical geometries of the cavity and the neck [6,7]. The classical approach approximates the HR as an equivalent spring-mass system with an end-correction length to take the spatial distribution effects into account. The wave propagation approach in both the duct and the HR has been investigated from a one-dimensional approach in preliminary to a multidimensional approach to account for the effect of nonplanar waves in the cavity and neck excited at the discontinuity area (the neck-cavity interface) [8,9].

Since the narrow-band behavior of HR is not practical for use in engineering applications, it is therefore that a broader noise attenuation band performance of the HR has attracted the attention of many researchers and engineers. A lot of efforts have been made in this area and could be found in numerous pieces of literature. Bradley [10,11] examined the propagation of time harmonic acoustic Bloch waves in periodic waveguides theoretically and experimentally. Seo and Kim [12] aimed to broaden the narrow band characteristics by combining many resonators and optimized the arrangement of resonators. Wang and Mak [13] investigated the wave propagation in a duct mounted with an array of identical resonators and presented theoretical methods of noise attenuation bandwidth prediction. Cai and Mak [14] proposed a noise control zone comprising the attenuation bandwidth or peak amplitude of a periodically ducted HR system. Their results indicated that the broader the noise attenuation band, the lower the peak attenuation amplitude. Cai et al. [15] suggested a modified ducted HR system by adding HRs on the available space in the transverse direction of the duct to improve the noise attenuation performance and fully utilizing an available space. Seo et al. [16] developed the prediction of the transmission loss of a silencer using resonator arrays at high sound pressure level. Richoux [17] addressed the propagation of high amplitude acoustic pules through a one-dimensional lattice of HR mounted on the waveguide and developed a new numerical method to consider the nonlinear wave propagation and the different mechanisms of dissipation. Langley [18] derived a closed-form expression for the wave transmission through disordered periodic waveguides of either length disorder or disorder in the inter-junction properties.

The periodic structure could provide a much broader noise attenuation band due to the coupling of the Bragg reflection and the resonance of HR. The number of HRs and the periodic distance are significant parameters for the achieved transmission loss. It should be noted that the noise attenuation capacity of every single HR in the system remains unchanged. It indicates that the number of HRs determines the noise attenuation performance of the whole system. However, a complete distance along the longitudinal direction of the duct for HR’s installation is sometimes unavailable in practical applications. Only several pieces of the duct may be available for the installation. The present work therefore concentrates on the acoustic performance of ducted HR system consist of several periodic parts. The Bragg theory and the transfer matrix method are used to investigate wave propagation in the duct. The acoustic performance of a periodic HR array and a HR array consist of several periodic parts are analyzed theoretically and numerically. The theoretical prediction results are verified by the Finite Element Method (FEM) simulation and show a good agreement with the FEM simulation results. The present study provides a practical way in noise control application of ventilation ductwork system by utilizing the advantage of periodicity and considering the unavailable completed duct length for HR’s installation.

## 2. Theoretical Analysis of a Periodic Helmholtz Resonator Array

### 2.1. A Single Helmholtz Resonator

The sound fields inside an HR are clearly multidimensional due to the discontinuities at the neck-cavity interface. The multidimensional modelling approach for a HR includes the effect of nonplanar waves excited at the discontinuity area. The classical approach approximates the HR as an equivalent spring-mass system with end-correction length to take the nonplanar wave effects into account. In view of the inherent narrow-band behavior of the HR, the multidimensional approach provides a more accurate HR design than the classical approach [19]. However, the main purpose here is to investigate the acoustic performance of the ducted HR system. It is therefore that the classical approach with end-correction length according to Ingard [6] is adopted in this study and is given as:

(1) $$Z_{r}=j(\omega \frac{\rho_{0}l_{n}^{\prime }}{S_{n}}-\frac{1}{\omega }\frac{\rho_{0}{c_{0}}^{2}}{V_{c}})$$

where $Z_{r}$ is the acoustic impedance of the HR, $\rho_{0}$ is air density, $c_{0}$ is the speed of sound in the air, $l_{n}^{\prime }$ and $S_{n}$ are the neck’s effective length and area respectively, $V_{c}$ is the cavity volume, ω is the circular frequency.

**Figure 1:**
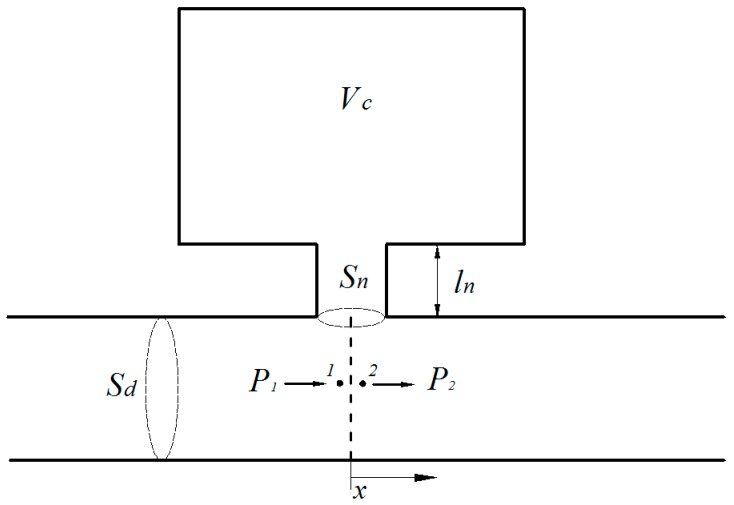
A single side-branch Helmholtz resonator.

A single side-branch HR is illustrated in Figure 1. Once the resonator impedance has been obtained, the transmission loss of the single side-branch HR can be determined by the four-pole parameter method [20,21] as:

(2) $$TL=20\log_{10}(\frac{1}{2}\left|2+\frac{\rho_{0}c_{0}}{S_{d}}\frac{1}{Z_{r}}\right|)$$

where $S_{d}$ is the cross-section area of the duct.

### 2.2. Theoretical Analysis of a Periodic Helmholtz Resonator Array

**Figure 2:**
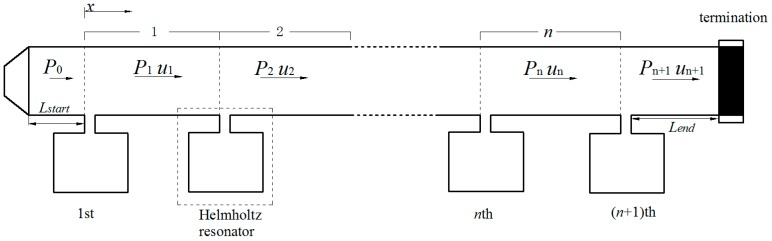
A periodic Helmholtz resonator array installed on the duct.

A periodic HR array installed on the duct is shown in Figure 2. A periodic unit is composed of a connection tube and a HR. The diameter of the HR’s neck is assumed to be negligible compared with the length of the connection tube in a periodic unit. It is therefore that the length of the connection tube is considered as the periodic distance. As low frequency range is the main concern in ventilation ductwork noise control, the frequency range considered in this paper is well below the cutoff frequency of the duct. Only planar wave is assumed to be exist in the duct propagation. The transfer matrix method is used to investigate wave propagation in the connection tube. The characteristics of sound in the *n*th unit could be described as sound pressure $p_{n}\left(x\right)$ and particle velocity $u_{n}\left(x\right)$. Assuming a time-harmonic disturbance in the form of $e^{j\omega t}$, both the sound pressure and the particle velocity are a combination of positive-*x* and negative-*x* directions and could be expressed as:

(3) $$p_{n}\left(x\right)=I_{n}e^{-jk\left(x-x_{n}-\omega t\right)}+R_{n}e^{jk\left(x-x_{n}+\omega t\right)}$$

(4) $$u_{n}\left(x\right)=\frac{I_{n}}{\rho_{0}c_{0}}e^{-jk\left(x-x_{n}-\omega t\right)}-\frac{R_{n}}{\rho_{0}c_{0}}e^{jk\left(x-x_{n}+\omega t\right)}$$

where *k* is the number of waves, $x_{n}=(n-1)d$ represents the local coordinates, *d* is the periodic distance, and $I_{n}$ and $R_{n}$ represent respective complex wave amplitudes. Considering the continuity condition of sound pressure and volume velocity at the point *x = nd* yields:

(5) $$\left[\begin{array}{c} I_{n+1}\\ R_{n+1} \end{array}\right]=\left[\begin{array}{cc} \exp(-jkd) & 0\\ 0 & \exp(jkd) \end{array}\right]\left[\begin{array}{cc} (1-\rho_{0}c_{0}/2S_{d}Z_{r}) & -\rho_{0}c_{0}/2S_{d}Z_{r}\\ \rho_{0}c_{0}/2S_{d}Z_{r} & (1+\rho_{0}c_{0}/2S_{d}Z_{r}) \end{array}\right]\left[\begin{array}{c} I_{n}\\ R_{n} \end{array}\right]=T\left[\begin{array}{c} I_{n}\\ R_{n} \end{array}\right]$$

T is the transfer matrix. It can be seen from Equation (5) that the characteristic of sound in arbitrary unit could be obtained once the initial sound pressure is given. Owing to the periodicity, Equation (5) could be rewritten in the form of Bloch wave theory [10] as:

(6) $$\left[\begin{array}{c} I_{n+1}\\ R_{n+1} \end{array}\right]=\exp(-jqd)\left[\begin{array}{c} I_{n}\\ R_{n} \end{array}\right]=T\left[\begin{array}{c} I_{n}\\ R_{n} \end{array}\right]$$

where q is the Bloch wave number and is allowed to be a complex value. According to Equation (6), the analysis of the periodic ducted HR system translates to an eigenvalue and its corresponding eigenvector issue. In general, the eigenvalue λ=exp(−jqd) describes the propagation property of a characteristic wave type, and its corresponding eigenvector defines the characteristic wave type. There are two solutions for λ: $\lambda_{1}$ and $\lambda_{2}$ with corresponding eigenvectors ${\left[v_{I1},v_{R1}\right]}^{T}$ and ${\left[v_{I2},v_{R2}\right]}^{T}$ respectively. Note that the determination of transfer matrix T is unit for a passive system [22]. The two relation of two solutions could be given as: $\lambda_{1}\lambda_{2}=1$. Assuming $\lambda_{1}$ describes the positive-*x* propagation, it means that $\left|\lambda_{1}\right|<1$, $\left|\lambda_{2}\right|>1$. Then Equation (6) could be rewritten in another form as:

(7) $$\left[\begin{array}{c} I_{n+1}\\ R_{n+1} \end{array}\right]=T\left[\begin{array}{c} I_{n}\\ R_{n} \end{array}\right]=T^{2}\left[\begin{array}{c} I_{n-1}\\ R_{n-1} \end{array}\right]=...=T^{n}\left[\begin{array}{c} I_{1}\\ R_{1} \end{array}\right]=A_{0}\lambda_{1}^{n}\left[\begin{array}{c} v_{I1}\\ v_{R1} \end{array}\right]+B_{0}\lambda_{2}^{n}\left[\begin{array}{c} v_{I2}\\ v_{R2} \end{array}\right]$$

The complex constants $A_{0}$ and $B_{0}$ could be achieved according to the boundary conditions. Assuming termination of the duct is anechoic, the reflection coefficient α=0 gives:

(8) $$\frac{R_{n+1}e^{jk\left(x-x_{n+1}+\omega t\right)}}{I_{n+1}e^{-jk\left(x-x_{n+1}-\omega t\right)}}=\frac{A_{0}{\lambda_{1}}^{n}v_{R1}e^{jkL_{end}}+B_{0}{\lambda_{1}}^{n}v_{R2}e^{jkL_{end}}}{A_{0}{\lambda_{1}}^{n}v_{I1}e^{-jkL_{end}}+B_{0}{\lambda_{1}}^{n}v_{I2}e^{-jkL_{end}}}=\alpha =0$$

The initial condition gives:

(9) $$\begin{array}{l} p_{0}={I_{0}e^{-jk\left(x+d\right)}+R_{0}e^{jk\left(x+d\right)}|}_{x=-L_{start}}\\ =(A_{0}{\lambda_{1}}^{-1}v_{I1}+B_{0}{\lambda_{2}}^{-1}v_{I2})e^{-jk\left(d-L_{start}\right)}+(A_{0}{\lambda_{1}}^{-1}v_{R1}+B_{0}{\lambda_{2}}^{-1}v_{R2})e^{jk\left(d-L_{start}\right)} \end{array}$$

Therefore, the average transmission of the system could be expressed as:

(10) $$\bar{T}\bar{L}=\frac{20}{n+1}\log_{10}\left|\frac{I_{0}}{I_{n+1}}\right|=\frac{20}{n+1}\log_{10}\left|\frac{A_{0}{\lambda_{1}}^{-1}v_{I1}+B_{0}{\lambda_{2}}^{-1}v_{I2}}{A_{0}{\lambda_{1}}^{n}v_{I1}+B_{0}{\lambda_{2}}^{n}v_{I2}}\right|$$

When the duct ends with an anechoic termination, no negative-*x* propagation wave exists in the last part of the duct. This indicates that $B_{0}=0$ is required in this situation. The average transmission loss of the system is then simplified as $\bar{T}\bar{L}=-20\log_{10}\left|\lambda_{1}\right|$. Equation (5) indicates that $\lambda_{1}$ is a function of the frequency, periodic distance and acoustic impedance of the HR.

The introduction of a periodic structure may help to achieve a wider noise attenuation band at the resonance frequency of the HR. The noise attenuation band of a periodic structure is induced physically by two mechanisms: the resonance of HR and the Bragg reflection. When the Bragg reflection frequency is intended to coincide with the designed resonance frequency, a broader noise attenuation band could be obtained. It is therefore that the periodic distance is chosen as $d=m\times \lambda_{0}/2$ (m is integer) to meet the requirement of coupling. In terms of practical application, the periodic distance is often chosen as $d=\lambda_{0}/2$ for the sake of the coupling of HR’s resonance and the first Bragg reflection to achieve a relatively broader noise attenuation band [23].

## 3. Theoretical Analysis of a Helmholtz Resonator Array Consist of Several Periodic Parts

**Figure 3:**
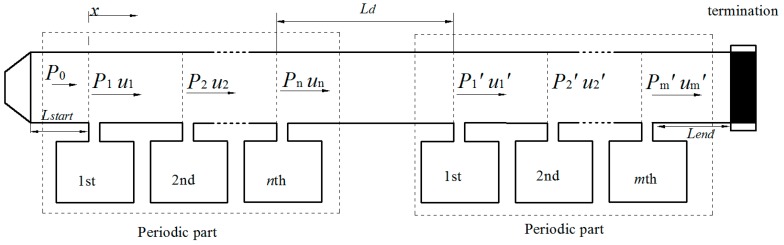
A ducted HR system consist of several periodic parts.

The periodic structure could provide a much broader noise attenuation band due to the coupling of the Bragg reflection and the HRs’ resonance. However, the noise attenuation capacity of every single HR in the system remains unchanged [14]. The transmission loss of the whole system is fairly dependent on the number of HRs. Besides, the complete distance along the longitudinal direction of the duct for HRs’ installation is sometimes unavailable in practical applications. Only several pieces of the duct are available for the installation. It is therefore that the ducted HR system consist of several periodic parts should be taken into account in practical applications. The present study concentrates on the acoustics performance of the ducted HR system consisting of two periodic parts, as illustrated in Figure 3. The periodic distance of the two periodic part remains the same. The length of the connection tube between two periodic parts is *L_d_*. The number of HRs installed in each periodic part is depended on the available length of the duct. As discussed above, the characteristics of sound wave propagation in these two periodic part could be described as the transfer matrix given in Equation (5). Once the periodic distance in each periodic part is chosen to be the same. It indicates that the eigenvalues $\lambda_{1}$ and $\lambda_{2}$ with corresponding eigenvectors ${\left[v_{I1},v_{R1}\right]}^{T}$ and ${\left[v_{I2},v_{R2}\right]}^{T}$ are also the same in these two periodic parts. It is therefore that the characteristics of sound wave propagation in these two periodic parts could be expressed as:

(11) $$\left[\begin{array}{c} I_{n}\\ R_{n} \end{array}\right]=T^{n-1}\left[\begin{array}{c} I_{1}\\ R_{1} \end{array}\right]=A_{0}\lambda_{1}^{n-1}\left[\begin{array}{c} v_{I1}\\ v_{R1} \end{array}\right]+B_{0}\lambda_{2}^{n-1}\left[\begin{array}{c} v_{I2}\\ v_{R2} \end{array}\right]$$

(12) $$\left[\begin{array}{c} I_{m}^{\prime }\\ R_{m}^{\prime } \end{array}\right]=T^{m-1}\left[\begin{array}{c} I_{1}^{\prime }\\ R_{1}^{\prime } \end{array}\right]=A_{0}^{\prime }\lambda_{1}^{m-1}\left[\begin{array}{c} v_{I1}\\ v_{R1} \end{array}\right]+B_{0}^{\prime }\lambda_{2}^{m-1}\left[\begin{array}{c} v_{I2}\\ v_{R2} \end{array}\right]$$

where *n* and *m* are the number of HRs installed in periodic parts respectively, $A_{0}^{\prime }$ and $B_{0}^{\prime }$ are also the complex constant related to the boundary conditions.

Only planar waves are assumed to exist in the duct propagation in the present study. The wave propagation in the connection tube of the two periodic parts can be given as:

(13) $$\left[\begin{array}{c} I_{1}^{\prime }\\ R_{1}^{\prime } \end{array}\right]=\left[\begin{array}{cc} \left(1-\frac{\rho_{0}c_{0}}{2S_{d}Z_{r}})\exp(-jkL_{d}\right) & \frac{\rho_{0}c_{0}}{2S_{d}Z_{r}}\exp(-jkL_{d})\\ \frac{\rho_{0}c_{0}}{2S_{d}Z_{r}}\exp(jkL_{d}) & \left(1+\frac{\rho_{0}c_{0}}{2S_{d}Z_{r}})\exp(jkL_{d}\right) \end{array}\right]\left[\begin{array}{c} I_{n}\\ R_{n} \end{array}\right]=\left[\begin{array}{cc} T_{11} & T_{12}\\ T_{21} & T_{11} \end{array}\right]\left[\begin{array}{c} I_{n}\\ R_{n} \end{array}\right]=T_{d}\left[\begin{array}{c} I_{n}\\ R_{n} \end{array}\right]$$

$T_{d}$ is the transfer matrix of the connection tube between two periodic parts. Combining Equations (11)–(13), the relation of complex constants of first periodic part and second periodic part could be given as:

(14) $$\{\begin{array}{c} A_{0}^{\prime }v_{I1}+B_{0}^{\prime }v_{I2}=T_{11}(A_{0}{\lambda_{1}}^{n-1}v_{I1}+B_{0}{\lambda_{2}}^{n-1}v_{I2})+T_{12}(A_{0}{\lambda_{1}}^{n-1}v_{R1}+B_{0}{\lambda_{2}}^{n-1}v_{R2})\\ A_{0}^{\prime }v_{R1}+B_{0}^{\prime }v_{R2}=T_{21}(A_{0}{\lambda_{1}}^{n-1}v_{I1}+B_{0}{\lambda_{2}}^{n-1}v_{I2})+T_{22}(A_{0}{\lambda_{1}}^{n-1}v_{R1}+B_{0}{\lambda_{2}}^{n-1}v_{R2}) \end{array}$$

Similar to the periodically ducted HR system, the initial condition in this situation gives the same equation as Equation (9). According to Equation (8), the end condition gives:

(15) $$\frac{R_{m}^{\prime }e^{jk\left(x-x_{m}+\omega t\right)}}{I_{m}^{\prime }e^{-jk\left(x-x_{m}-\omega t\right)}}=\frac{A_{0}^{\prime }{\lambda_{1}}^{m-1}v_{R1}e^{jkL_{end}}+B_{0}^{\prime }{\lambda_{1}}^{m-1}v_{R2}e^{jkL_{end}}}{A_{0}^{\prime }{\lambda_{1}}^{m-1}v_{I1}e^{-jkL_{end}}+B_{0}^{\prime }{\lambda_{1}}^{m-1}v_{I2}e^{-jkL_{end}}}=\alpha$$

where $x_{m}=(n-1)d+L_{d}$ represents the local coordinates in the second periodic part. 

Therefore, the average transmission of the system could be expressed as:

(16) $$\bar{T}\bar{L}=\frac{20}{n+m}\log_{10}\left|\frac{I_{0}}{I_{m}^{\prime }}\right|=\frac{20}{m+n}\log_{10}\left|\frac{A_{0}{\lambda_{1}}^{-1}v_{I1}+B_{0}{\lambda_{2}}^{-1}v_{I2}}{A_{0}^{\prime }{\lambda_{1}}^{m-1}v_{I1}+B_{0}^{\prime }{\lambda_{1}}^{m-1}v_{I2}}\right|$$

The complex constants $A_{0}$, $B_{0}$, $A_{0}^{\prime }$ and $B_{0}^{\prime }$ could be derived by combining the boundary conditions (Equations (9) and (15)) and their interrelationship (Equation (14)). When the duct ends with an anechoic termination (α = 0), $B_{0}^{\prime }=0$ is similarly compulsory.

It should be noted that the above theoretical analysis approach could also be applied to a ducted HR system consisting of several periodic parts (*n* parts). It indicates that the total number of complex constants is 2*n.* According to the initial condition and the end condition, Equations (9) and (15) could be derived, respectively. The transfer matrix of connection tube between each periodic parts could be obtained according to Equation (13). It means that the number of equations described the relation of complex constants in each periodic part equals 2(*n* − 1)*.* Then the numbers of complex constants to be solved and independent equations are both 2*n.* The complex constants in the first and last periodic part could be solved by a set of equations. Therefore, the average transmission of the system could be achieved by Equation (16).

## 4. Results and Discussion

The periodic HR array installed on the ducted and the ducted HR system consist of two periodic parts are illustrated in Figure 2 and Figure 3 respectively. The geometries of the HR used in this study are: neck area $S_{n}=4\pi$ cm^2^, $l_{n}=2.5$ cm^2^, and cavity volume $V_{c}=101.25\pi$ cm^3^. The cross-section area of the main duct is $S_{d}=36$ cm^2^. The anechoic termination is applied at the end of the duct in both systems to avoid reflected waves from the termination. An oscillating sound pressure at a magnitude of $P_{0}=1$ is applied at the beginning of the duct as the initial boundary condition. The three-dimensional FEM simulation is used to validate the theoretical predictions. A detailed description of the FEM for time-harmonic acoustics in the present study, which are governed by the Helmholtz equation, can be found in numerous references and could be considered as a reliable validation method [24]. All models in this study are divided into more than 150,000 tetrahedral elements by mesh. The mesh divides each neck and cavity into more 3000 and 6000 tetrahedral elements respectively. With the purpose of the accuracy, a fine mesh spacing of less than 6 cm is adopted for the models. The maximum element with a side length of around 5.8 cm could be found in the duct domain; the minimum element is observed in the neck-cavity interface domain with a side length of around 2.1 mm.

### 4.1. Validation of the Theoretical Predicitons of a Periodic Helmholtz Resonator Array

**Figure 4:**
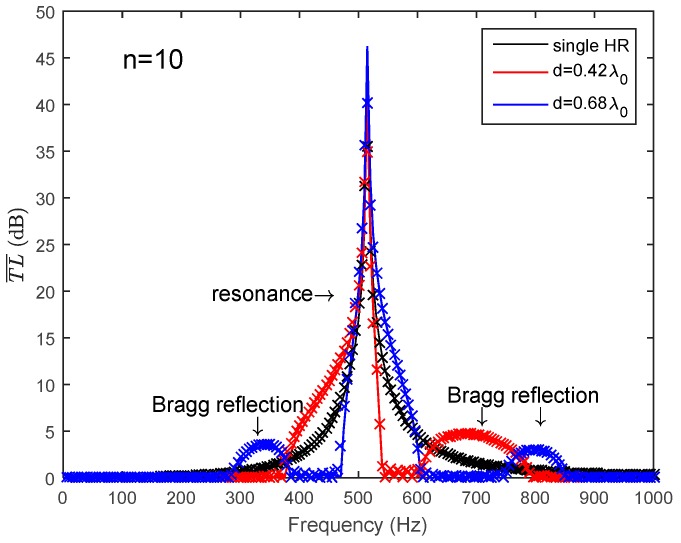
The noise attenuation band of a periodic HR array due to Bragg reflection and HR’s resonance separately (lines represents the theoretical predictions, and dotted crossed represent the FEM simulation results).

The average transmission loss of a periodic HR array is expressed as $\bar{T}\bar{L}=-20\log_{10}\left|\lambda_{1}\right|$. $\lambda_{1}$ is a function of the frequency, periodic distance and acoustic impedance of the HR. For a certain HR used in this paper, it means that acoustic impedance of the HR is determined by Equation (1). Then, the shape of the $\bar{T}\bar{L}$ is only dependent on the periodic distance in the frequency domain. The number of HRs installed on the duct is *n* = 10 here. When the periodic HR array chooses $d=0.42\lambda_{0}/2$ or $d=0.68\lambda_{0}/2$ as the periodic distance, it can be seen from Figure 4 that the HRs’ resonance and the Bragg reflection have separated effects on the noise attenuation band. A broader noise attenuation band will not be achieved without the coupling effects. The comparison of the analytical predictions and the FEM simulation results are also illustrated in Figure 4. The solid lines represent the analytical predictions and the dotted crosses represent the FEM simulation results. It can be seen that the predicted results show a good agreement with the FEM simulation results.

**Figure 5:**
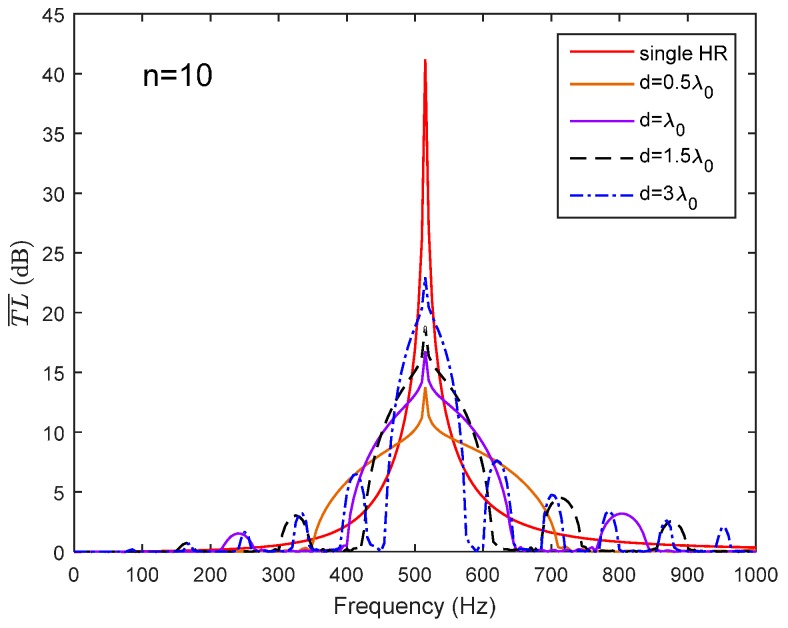
The noise attenuation band of a periodic HR array due to coupling of Bragg reflection and HR’s resonance frequency.

**Figure 6:**
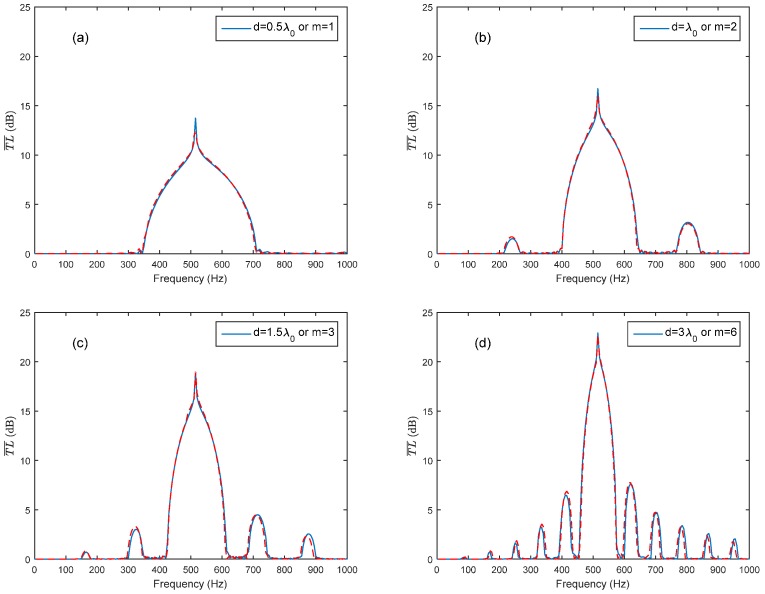
Comparison of theoretical predictions and the FEM simulation in respect of different periodic distances (solid lines represent the theoretical predictions, and dashed lines represent the FEM simulation results): (**a**) periodic distance $d=\lambda_{0}/2$; (**b**) periodic distance $d=\lambda_{0}$; (**c**) periodic distance $d=1.5\lambda_{0}$; (**d**) periodic distance $d=3\lambda_{0}$.

In order to obtain a broader noise attenuation band, the Bragg reflection is intended to coincide with the HR’s resonance frequency. It is therefore that the periodic distance is chosen as $d=m\times \lambda_{0}/2$ (m is an integer) to meet the requirement of coupling. Figure 5 exhibits a broader noise attenuation band due to the coupling of the Bragg reflection and HR’s resonance. It can be seen in Figure 5 that with the increasing in periodic distance (integer m increases from 1 to 6), the width of noise attenuation band decreased. For the sake of a broader noise attenuation band, the periodic distance is often selected as $d=\lambda_{0}/2$ to meet the requirement of the coupling of HR’s resonance and the first Bragg reflection in practical applications. Figure 6 compares the theoretical predictions with the FEM simulation results in respect of different periodic distances (or different integer *m*), and the predicted results fit well with the FEM simulations results.

### 4.2. Validation of the Theoretical Predicitons of a Helmholtz Resonator Array Consist of Serveral Periodic Parts

**Figure 7:**
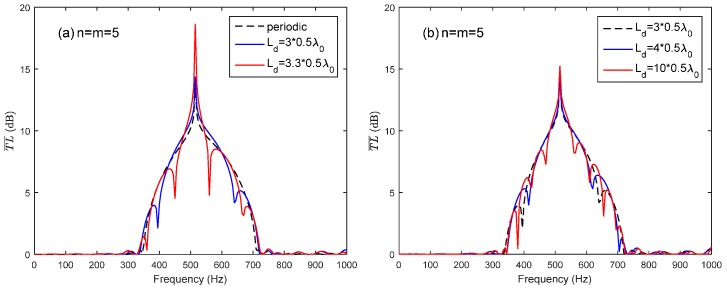
The average transmission loss of the HR array with respect to different connection tube lengths *L_d_* between the two periodic parts: (**a**) the periodic one versus two arrays with $L_{d}=3\times 0.5\lambda_{0}$ and $L_{d}=3.3\times 0.5\lambda_{0}$ respectively; (**b**) three arrays with $L_{d}=3\times 0.5\lambda_{0}$, $L_{d}=4\times 0.5\lambda_{0}$, and $L_{d}=10\times 0.5\lambda_{0}$ respectively.

**Figure 8:**
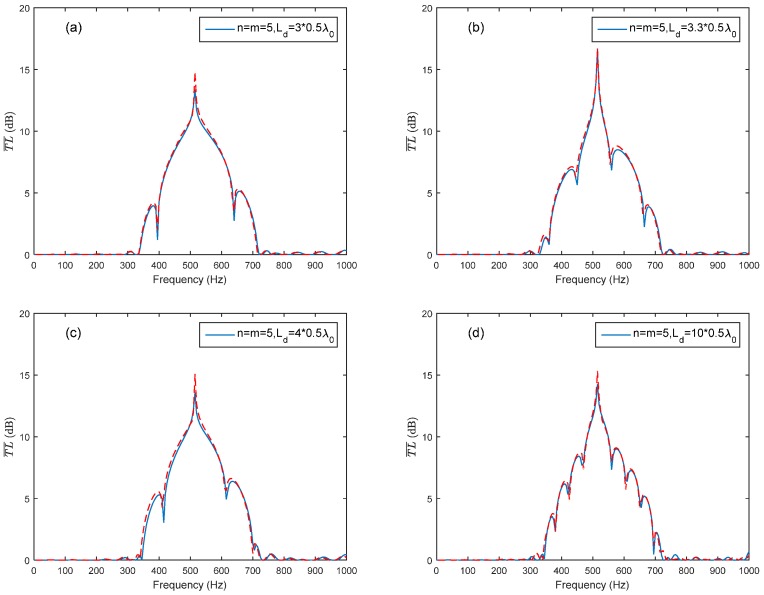
Comparison of theoretical predictions and the FEM simulation with respect to different connection tube lengths *L_d_* between the two periodic parts (solid lines represent the theoretical predictions, and dashed lines represent the FEM simulation results): (**a**) *n* = *m* = 5, $L_{d}=3\times 0.5\lambda_{0}$; (**b**) *n* = *m* = 5, $L_{d}=3.3\times 0.5\lambda_{0}$; (**c**) *n* = *m* = 5, $L_{d}=4\times 0.5\lambda_{0}$; (**d**) *n* = *m* = 5, $L_{d}=10\times 0.5\lambda_{0}$.

By taking the advantage of the coupling of the Bragg reflection and the HR’s resonance, the periodic structure could provide a broader noise attenuation band. However, the complete distance along the longitudinal direction of the duct is sometimes unavailable for HR installation in practical applications. Only several pieces of the duct may be available for the installation. A HR array consisting of two periodic parts is sketched in Figure 4. The periodic distance of the two periodic parts is both adopted as $d=\lambda_{0}/2$ to meet the coupling requirement of the HR’s resonance and the first Bragg reflection for the sake of a broader noise attenuation band. The total number of HRs used in the HR array consist of two parts is 10 (*n + m* = 10). Two arrangement cases are investigated in this study: *n* = *m* = 5 and *n* = 3, *m* = 7. Figure 7a compares $\bar{T}\bar{L}$ of case (*n* = *m* = *5*) with different *L_d_* ($L_{d}=3\times 0.5\lambda_{0},3.3\times 0.5\lambda_{0}$) to periodic ducted HR system (ten HRs and $L_{d}=0.5\lambda_{0}$). When the $L_{d}$ is chosen as integral multiple of half-wavelength of HR’s resonance frequency ($L_{d}=3\times 0.5\lambda_{0}$), it has less effects on the noise attenuation band than $L_{d}=3.3\times 0.5\lambda_{0}$. The reason is that the Bragg inflection coincides with the HR’s resonance by considering the wave propagation from the last HR in the first periodic part to the first HR in the second periodic part when $L_{d}=3\times 0.5\lambda_{0}$ rather than $L_{d}=3.3\times 0.5\lambda_{0}$. Compared with the periodic HR array, the HR array consisting of two periodic parts with a connection tube length $L_{d}=3\times 0.5\lambda_{0}$ also has acceptable noise attenuation band bandwidth, especially at the designed resonance frequency. However, with the increasing value of $L_{d}$, the Bragg reflection effects results in a more fluctuation noise attenuation band instead of the a dome-like band, as shown in Figure 7b. A good agreement between the theoretical predicted $\bar{T}\bar{L}$ and the FEM simulation results can be seen in Figure 8.

**Figure 9:**
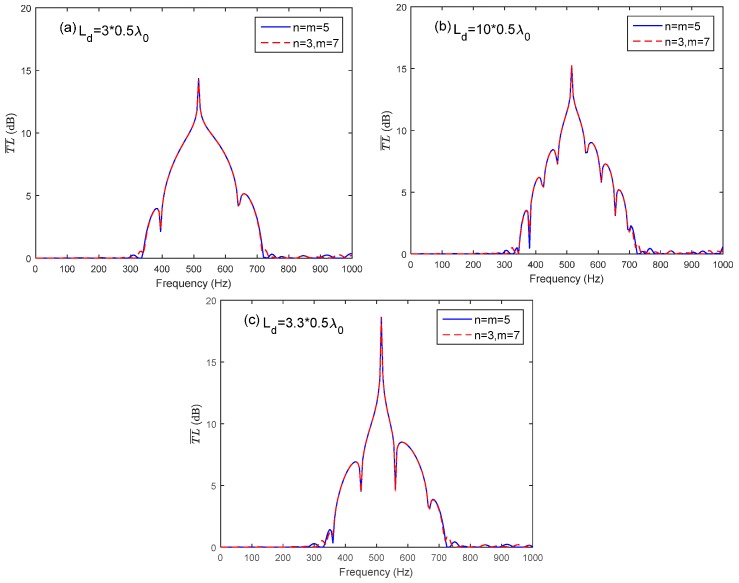
Comparison of two arrangement cases (*n = m =* 5 and *n* = 3, *m* = 7) with identical number of HRs and connection tube length *L_d_*: (**a**) two different arrangements with same $L_{d}=3\times 0.5\lambda_{0}$; (**b**) two different arrangements with same $L_{d}=10\times 0.5\lambda_{0}$; (**c**) two different arrangements with same $L_{d}=3.3\times 0.5\lambda_{0}$.

**Figure 10:**
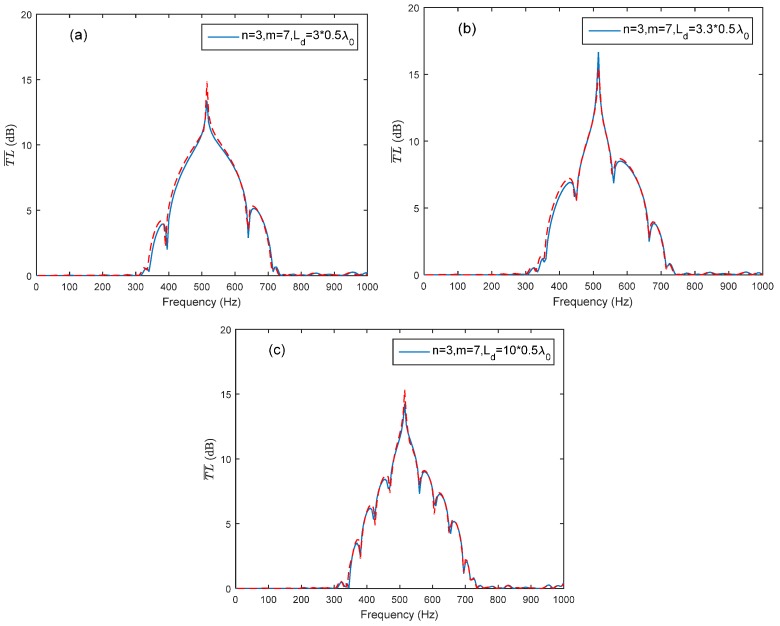
The average transmission loss of the HR array consist of two periodic parts (*n* = 3, *m* = 7) with different connection tube length *L_d_* (solid lines represent the theoretical predictions, and dashed lines represent the FEM simulation results): (**a**) $L_{d}=3\times 0.5\lambda_{0}$; (**b**) $L_{d}=3.3\times 0.5\lambda_{0}$; (**c**) $L_{d}=10\times 0.5\lambda_{0}$.

The comparison of two arrangement cases (*n* = *m* = 5 and *n =* 3, *m =* 7) with identical number of HRs and connection tube length *L_d_* is illustrated in Figure 9. The average transmission loss of the two arrangement cases with identical *L_d_* is nearly the same. This means that the arrangement of HRs in periodic parts has no effect on the average transmission loss. The connection tube length and the periodic distance are the significant parameters in ventilation ductwork noise control. The results provide a useful way in noise control application by utilizing the advantage and considering the insufficient duct length for a periodic HR array. The theoretical predictions fit well with the FEM simulation results, as shown in Figure 10.

## 5. Conclusions

A periodic HR array could provide a broader noise attenuation band due to the coupling of the Bragg reflection and the HRs’ resonance. However, the transmission loss achieved by a periodic ducted HR system is mainly depended on the number of HRs. The number of HRs is restricted by the available space in the longitudinal direction of the duct. The full distance along the longitudinal direction of the duct is sometimes unavailable for HR installation in practical applications. Only several pieces of the duct may be available for the installation. The acoustic performance of a periodic HR array and a HR array consist of several periodic parts are analyzed theoretically and numerically. For a periodic HR array, an appropriate periodic distance can broaden the noise attenuation band compared to a single resonator due to the coupling of the Bragg reflection and the HRs’ resonance. For a HR array consist of several periodic parts, the comparison of two arrangement cases with different connection tube lengths *L_d_* between the two periodic parts shows that the arrangement of HRs on periodic parts has no effect on the average transmission loss. The average transmission loss is only related to the connection tube length. When the $L_{d}$ is chosen as integral multiple of half-wavelength of HR’s resonance frequency, it has less effects on the noise attenuation band than the non-integral multiple of half-wavelength. The reason is that the Bragg inflection coincides with the HRs’ resonance by considering the wave propagation from the last HR in the first periodic part to the first HR in the second periodic part when integral multiple is chosen rather than non-integral multiple. However, with the increasing of $L_{d}$, the Bragg reflection effects results in a more fluctuation noise attenuation band instead of the a dome-like noise attenuation band of a periodic HR array. The results indicate that the shorter the connection tube length, the less effect on the transmission loss. The theoretical prediction results show a good agreement with the FEM simulation results. The present study provides a useful method for noise control application of ventilation ductwork systems by utilizing the advantage of the periodicity to broaden the noise attenuation band and considering the insufficient duct length for a pure periodic HR array. It has a potential application in actual noise control application with the limitation of available completed duct length for HRs’ installation.
